# The association between blood groups, Rhesus factors, body mass index and obesity among pregnant women at Gadarif Maternity Hospital, Eastern Sudan

**DOI:** 10.1186/s12884-023-06125-z

**Published:** 2023-11-17

**Authors:** Amal O Bashir, Ahmed Ali Hassan, EL Bagir Mahdi, Gamal K Adam, Nadiah AlHabardi, Ishag Adam

**Affiliations:** 1 Faculty of Public Health and Health Informatics, University of Umm Al Qura, Mekkah, Saudi Arabia; 2 Faculty of Medicine, University of Khartoum, Khartoum, Sudan; 3 Department of Obstetrics and Gynecology, Faculty of Medicine, Umm Al-Qura University, Mecca, Saudi Arabia; 4 Faculty of Medicine, Gadarif University, Gadarif, Sudan; 5 Department of Obstetrics and Gynecology, Unaizah College of Medicine and Medical Sciences, Qassim University, Unaizah, 51911 Saudi Arabia

**Keywords:** Blood group, Obesity, Body mass index, Pregnant women, Risk factors, Gadarif, Eastern Sudan

## Abstract

**Background:**

The existing evidence regarding the link between blood groups and obesity remains inconclusive, and there is a noticeable lack of data on the potential association between blood groups and obesity during pregnancy. Consequently, this study aimed to investigate the association between blood groups, body mass index (BMI), and obesity among pregnant women receiving care at Gadarif Maternity Hospital in eastern Sudan.

**Methods:**

This cross-sectional study was conducted in eastern Sudan during the period from April to September 2022. A questionnaire was employed to gather sociodemographic information from pregnant women. BMI was computed based on weight and height. Blood groups determinations were made using the agglutination method which is commonly used in the study’s region. Multinominal and multiple linear regression analyses were performed, and adjusted for covariates in the regression models.

**Results:**

Eight hundred and thirty-three pregnant women were enrolled with a median (interquartile range, IQR) gestational age of 10.0 (9.3‒11.0) weeks. The median (IQR) BMI of the women was 26.3(24.2‒29.4) kg/m^2^. Of these women, 11(1.3%) were underweight, 268(32.2%) were of normal weight, 371(44.5%) were overweight, and 183(22.0%) were obese. One hundred eighty-three (22.0%) women had blood group A, 107 (12.8%) had blood group B, 56 (6.7%) had blood group AB, and 487(58.5%) had blood group O. While 798 (95.8%) of the women were Rhesus factor positive, only 35 (4.2%) were Rhesus factor negative. Multinominal regression showed that only urban residency (adjusted odds ratio, AOR = 2.46, 95% confidence interval, CI = 1.47‒4.13) was associated with overweight. Blood groups and Rhesus factors were not associated with overweight. Age (AOR = 1.06, 95% CI = 1.01‒1.11), urban residence (AOR = 2.46, 95%, CI = 1.47‒4.13), and blood group O (AOR = 1.60, 95%, CI = 1.06‒2.40), were associated with obesity. Rhesus factors were not associated with obesity. In the multiple linear regression, age (coefficient = 0.07, P = 0.028), gravidity (coefficient = 0.25, P = 0.014), urban residence (coefficient = 1.33, P = 0.001), and blood group O (coefficient = 0.68, P = 0.035) were associated with BMI.

**Conclusions:**

Blood group O was associated with obesity and high BMI among pregnant women in eastern Sudan. Rhesus factors were not associated with obesity.

## Introduction

According to the World Health Organization (WHO), obesity is a major global health problem, and its prevalence nearly tripled between 1975 and 2016 to 11% of men and 15% of women [1]. Pregnant women are especially prone to obesity in many countries, including Africa [2–4]. Previous studies from different countries including Africa have shown that maternal obesity is associated with several adverse effects, such labor induction [5], caesarean delivery [6, 7], macrosomia [6], gestational hypertension [8], gestational diabetes [9], and anemia [3]. Moreover, the offspring of obese mothers have an increased risk of developing childhood obesity, cardiovascular diseases, diabetes mellitus, and stroke [10].

Several factors, including age [4], education [4], and parity [11], are associated with obesity among pregnant women. Blood groups have also been linked to obesity. It has been reported that blood groups are associated with adult hypertension (blood group B) [12], preeclampsia (blood group O) [13], *Helicobacter pylori* (*H. pylori*) infection in pregnant women (blood group O) [14], placental malaria infection (group O) [15], and coronavirus disease (COVID-19) (blood group O) [16]. However, previous studies have reported inconsistent findings regarding the association between blood groups and obesity. For example, studies have demonstrated links between obesity in young females and blood group O [17], obesity in adult women and blood groups O, B [18], and A [19], and obesity in adults and blood group B [12]. However, other studies have reported that blood groups were not associated with obesity in healthy adolescents [20], medical students [21], and young adults [22]. To the best of our knowledge, no previous studies have specifically examined the association between blood groups and obesity during pregnancy. Such knowledge could aid in early recognition of women risk of obesity based on their blood groups.

In Sudan, there is a high prevalence of maternal obesity in different regions, which has a negative impact on maternal and perinatal health [3, 4]. In fact, about one-third of Sudanese pregnant women have been diagnosed as obese [3]. Importantly, women who are overweight or obese before or during pregnancy are more likely to exhibit micronutrient deficiencies, including iron deficiency during pregnancy, which can lead to poor maternal and perinatal outcomes [23–25]. A recent study showed that obese pregnant Sudanese women had lower levels of serum iron, folate, and vitamin B12 [24, 25]. Thus, the current study aimed to investigate the association between blood groups, Rhesus factors, BMI, and obesity among pregnant women at Gadarif Maternity Hospital in eastern Sudan.

## Methods

### Study area and design

A cross-sectional study was conducted involving pregnant women who received antenatal care at Gadarif Maternity Hospital in eastern Sudan from April to September 2022. It is a referral hospital providing maternity care to the entire state of Gadarif. The capital city, also named Gadarif, is located in eastern Sudan about 400 km from the capital, Khartoum, on the Ethiopian border. Gadarif is one of the 18 states in Sudan and has a total population of 2.5 million inhabitants, which includes members of all tribes from all regions of Sudan [26].

### Study participants

During the study period, every pregnant woman who received antenatal care was approached by trained medical officers. The purpose of the study and the ethical issues were explained to eligible women with confirmed pregnancies by the officers.

### Inclusion criteria

Women who attended Gadarif Maternity Hospital for antenatal care in early pregnancy (< 14 weeks of gestation), had a singleton pregnancy, gave informed written consent, were apparently healthy, and who had no known illness were eligible to participate in the study.

### Exclusion criteria

Women who refused to give consent to participate in the study, those who were in their 2nd or 3rd trimesters (≥ 14 weeks of gestation), those with diabetes mellitus, hypertension, thyroid disease, renal disease, liver disease, or any other chronic disease, those with intrauterine fetal demise, and those who were severely ill were excluded from the study.

### Data collection

A questionnaire was used during face-to-face interviews to gather the data. The questionnaire was based on similar previous studies [13–16].Questions focused on sociodemographic information, including maternal age in years, place of residence (rural or urban), maternal education (< secondary or ≥ secondary), and occupation (housewives or employed), as well as clinical and obstetrical information, such as gravidity and gestational age in weeks (based on the last menstrual cycle and confirmed by ultrasound). Two female medical officers were trained by the investigators to gather the data through face-to-face interviews. Before data collection began, the questionnaire was tested among 15 mothers (not included in the final analysis), and all necessary corrections were made.

### Anthropometric measurements

Each woman’s weight was measured in kilograms (kg) using well-calibrated scales, which were adjusted to zero before each measurement. The women stood with minimal movement, hands by their sides, and shoes and excess clothing removed. Then, their height was measured in centimeters (cm) in a standing position with their backs against a wall and feet together. Body mass index (BMI) was computed as the weight in kg divided by the square of the height in meter (m) (kg/m^2^) [27]. Further, BMI was grouped according to the WHO classification, which encompasses obesity during pregnancy as well as in the general population: underweight (< 18.5 kg/m^2^), normal weight (18.5–24.9 kg/m^2^), overweight (25.0–29.9 kg/m^2^), or obese (≥ 30.0 kg/m^2^) [27].

### Blood analysis

Blood samples (2 ml) were collected under aseptic conditions from each participant for blood group analysis. Maternal blood groups were determined using the manual agglutination method (blood groups and Rhesus factors). After implementing all necessary safety precautions, a single drop from each 2-ml blood sample was placed into four round-bottom tubes. Subsequently, a drop of monoclonal anti-A, anti-B, and monoclonal/polyclonal anti-D reagents was added to each tube, followed by thorough mixing over a 2.5-cm area with gentle rocking motions. Agglutination reactions for ABO blood groups were immediately observed and recorded, while agglutination reactions for the Rhesus factor were recorded after a two-minute interval. The presence of agglutination indicated a positive result, signifying the presence of the corresponding antigen. Conversely, the absence of agglutination indicated a negative result, that is, absence of the corresponding antigen, as described in previous studies [15, 28]. Although the agglutination method is commonly used in limited resource settings such as eastern Sudan (simple, rapid, and low cost), however, the sensitivity of this method is low and can be easily affected by several factors; therefore cautions should be taken, especially in blood transfusion [29].

### Sample size calculation

OpenEpi Menu was used to compute the desired sample size [30]. A sample size of 833 pregnant women was deemed appropriate for the total study, and based on previous reports from Sudan, we assumed that 25.0% of these women would be obese (obese-to-non-obese ratio = 1:4) [31].Moreover, 43.7% of pregnant women in eastern Sudan have blood group O [15]. Therefore, we assumed that 45.0% and 58.0% of the normal weight and obese women would have blood group O, respectively. This sample size was calculated to detect a difference of 5% at *α* = 0.05, with a power of 80%.

### Statistical analysis

Data were entered in the computer using the IBM Statistical Package for the Social Sciences® (SPSS®) for Windows version 22.0 (SPSS Inc., New York, United States). The proportions were expressed as frequencies (%). The continuous data were evaluated for normality using the Shapiro–Wilk test and were non-normally distributed (age, gravidity, and BMI). The non-normally distributed data were expressed as medians (interquartile range: IQR). An adjusted regression analysis (multinominal and linear) was performed with overweight and obesity (for multinominal regression) and BMI (for linear regression) as the dependent variables. Sociodemographic (age, residence, educational level, and occupation) and clinical and obstetrical variables (gravidity, blood groups, and Rhesus factors) were independent variables.

Adjusted odds ratios (AORs), 95% confidence intervals (CIs), coefficients, and standard errors were calculated as they were applied. A two-sided P-value of < 0.05 was considered statistically significant.

## Results

### General characteristics

**Fig. 1:**
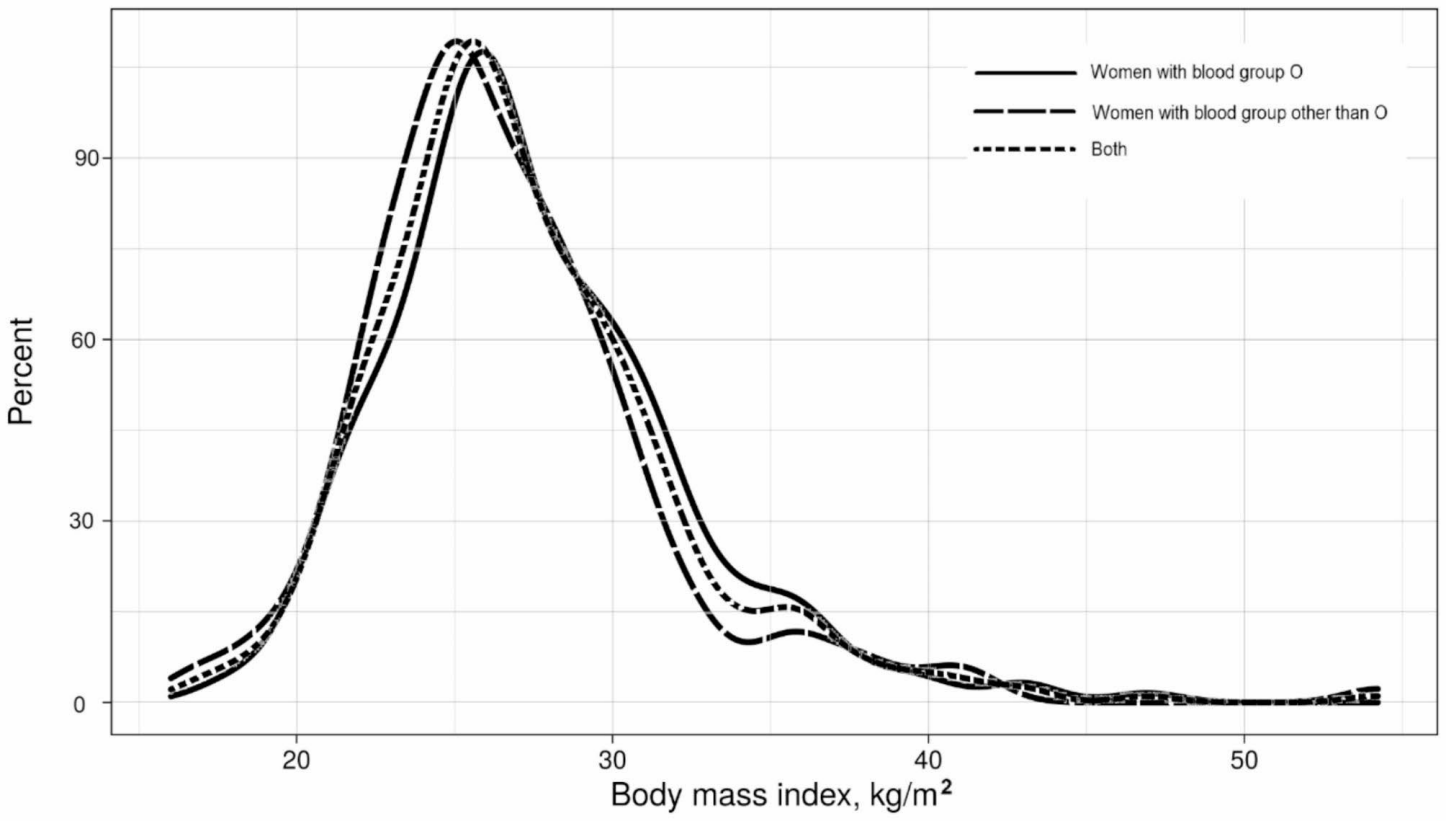
Kernel density plot of height and body mass index among pregnant women in eastern Sudan, 2022

Eight hundred and thirty-three pregnant women with a median (IQR) gestational age of 10.0(9.3‒11.0) weeks were enrolled in the study. The median (IQR) age of the 833 enrolled women was 24(20.0‒29.0) years, and the median gravidity was 2(1‒4). Of the women, 205(24.6%) had an education level ≥ secondary level, and 789 (94.7%) were housewives. The median (IQR) BMI was 26.3(24.2‒29.4) kg/m^2^. From the total 833, 11(1.3%) were underweight, 268(32.2%) were of normal weight, 371(44.5%) were overweight, and 183(22.0%) were obese, (Fig. 1).

Of the 833 participants, 183 (22.0%) had blood group A, 107(12.8%) had blook group B, 56(6.7%) had blood group AB, and 487(58.5%) had blood group O. While 798 (95.8%) women were Rhesus factor positive, only 35(4.2%) women were Rhesus negative (Fig. 1; Table 1).

**Table 1:** Sociodemographic and clinical characteristics of pregnant women in eastern Sudan, 2022

Variables	Median		Interquartile range
Age, years		24.0	20.0‒29.0
Gravidity		2	1‒4
Body mass index, kg/m^2^		26.3	24.2‒29.4
**Frequency (proportions)**			
Residence	Urban	234	28.1
	Rural	599	91.9
Education level	≥ secondary	205	24.6
	< secondary	628	75.4
Occupation	Housewives	789	94.7
	Employed	44	5.3
Body mass index Groups	Underweight	11	1.3
	Normal weight	268	32.2
	Overweight	371	44.5
	Obese	183	22.0
Blood groups	A	183	22.0
	B	107	12.8
	AB	56	6.7
	O	487	58.5
Rhesus factor	Positive	798	95.8
	Negative	35	4.2

### Factors associated with body mass index

**Fig. 2:**
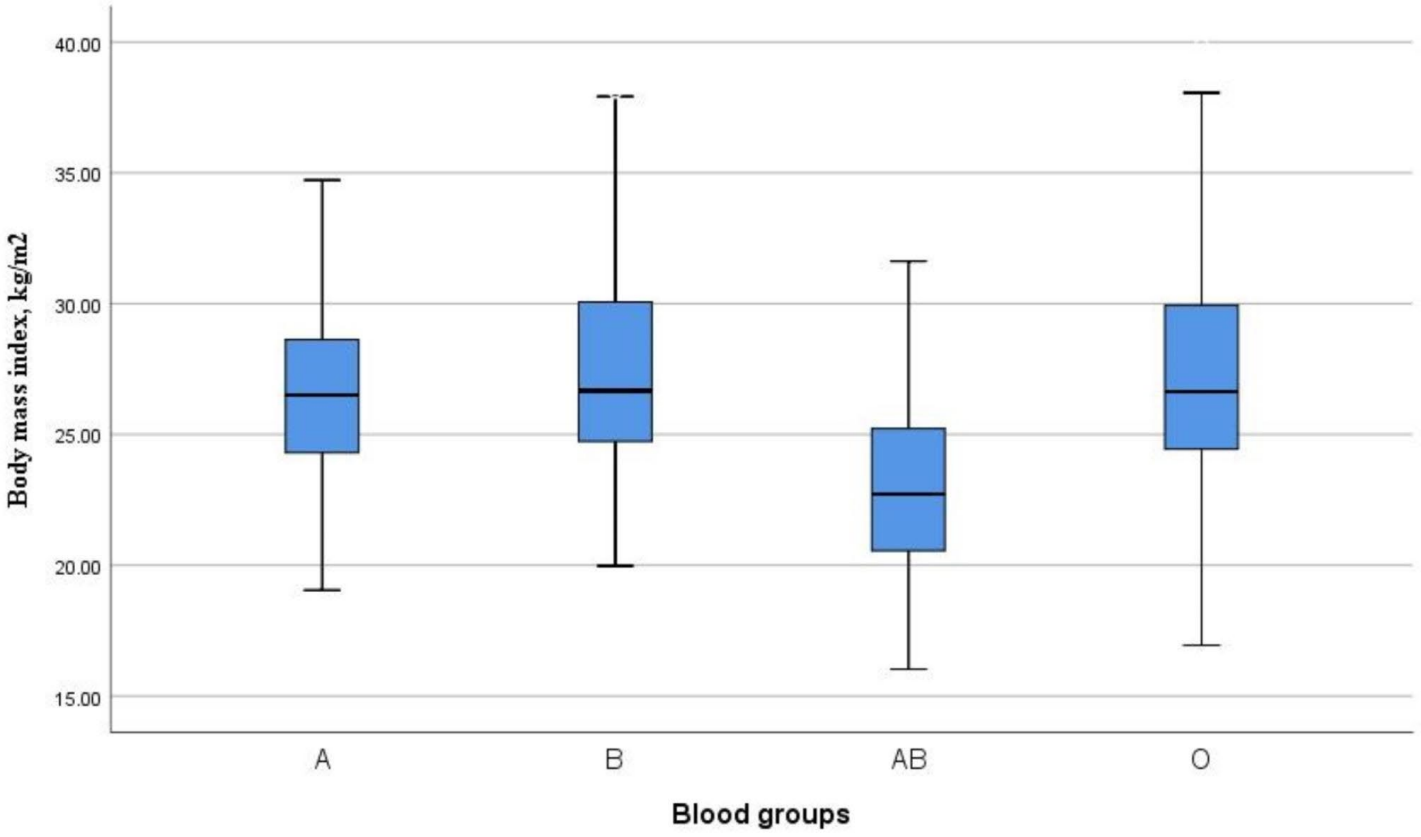
Comparison of body mass index by blood group among pregnant women in eastern Sudan, 2022

Obese women had significantly higher age and gravidity. Moreover, a significantly higher number of obese women had an urban residence, were employed, and had blood group O. There was no significant difference in education level or Rhesus factors between women in the different BMI groups (Table 2). Compared with women with other blood groups, women with blood groups B and O had a significantly BMI. Women with blood group O had a significantly higher BMI compared with women with blood groups other than O (P = 0.006, Fig. 2).

**Table 2:** Comparison of sociodemographic and clinical factors in pregnant women in eastern Sudan, 2022

Variables	Underweight (11)		Normal weight (268)	Overweight (371)	Obese (183)	P
**Median (interquartile range)**						
Age, years	25.0(18.0‒28.0)		22.0(18.0‒26.7)	24.0(20.0‒29.0)	26.0(22.0‒32.0)	< 0.001
Gravidity		1(1‒3)	2(1‒3)	2(1‒4)	3(1‒5)	< 0.001
**Frequency (proportions)**						
Residence	Urban	2(18.2)	44(16.4)	120(32.3)	68(37.2)	< 0.001
	Rural	9(81.2)	224(83.6)	251(67.7)	115(62.8)	
Education level	≥ secondary	2(18.2)	50(18.7)	102(27.5)	51(27.9)	0.045
	< secondary	9(81.8)	218(81.3)	269(72.5)	132(72.1)	
Employment	Housewives	11(100)	259(96.6)	354(95.4)	165(90.2)	0.014
	Employed	0(0)	9(3.4)	17(4.6)	18(9.8)	
Blood groups	A	0(0)	62(23.1)	90(24.3)	31(16.9)	< 0.001
	B	0(0)	29(10.8)	49(13.2)	29(15.8)	
	AB	7(63.6)	33(12.3)	13(3.5)	3(1.6)	
	O	4(36.4)	144(53.7)	219(59.0)	120(65.6)	
Rhesus factor	Positive	10(90.9)	258(96.3)	354(95.4)	176(96.2)	0.832
	Negative	1(9.1)	10(3.7)	17(4.6)	7(3.8)	

Multinominal regression showed that only urban residency (AOR = 2.46, 95% CI = 1.47‒4.13) was associated with overweight. No association was found between blood groups and Rhesus factors and overweight. Age (AOR = 1.06, 95% CI = 1.01‒1.11), urban residence (AOR = 2.46, 95%, CI = 1.47‒4.13), and blood group O (AOR = 1.60, 95%, CI = 1.06‒2.40) were associated with obesity. Rhesus factors were not associated with obesity (Table 3).

**Table 3:** Multinominal analysis of the factors associated with overweight and obesity among pregnant women in eastern Sudan, 2022

Variables		Overweight		Obese	
		OR (95% CI)	P	OR (95% CI)	P
Age, years		1.03(0.99‒1.07)	0.075	1.06 (1.01‒1.11)	0.014
Gravidity		1.01(0.92‒1.12)	0.718	1.11(0.99‒1.25)	0.058
Residence	Urban	2.11(1.35‒3.29)	0.001	2.46(1.47‒4.13)	0.001
	Rural	Reference			
Education level	≥ secondary	Reference			0.799
	< secondary	1.27(0.80‒2.01)	0.296	1.07(0.61‒1.90)	
Occupation	Housewives	References			0.437
	Employed	0.63(0.25‒1.58)	0.329	1.46(0.55‒3.84)	
Blood groups	Other than O	Reference			0.022
	O	1.32 (0.90‒1.92)	0.143	1.60 (1.06‒2.40)	
Rhesus factor	Positive	Reference			0.765
	Negative	0.69(0.27‒1.75)	0.436	0.85(0.29‒2.43)	

OR, odds ratio, CI, confidence interval

In the multiple linear regression, age (coefficient = 0.07, P = 0.028), gravidity (coefficient = 0.25, P = 0.014), urban residence (coefficient = 1.33, P = 0.001), and blood group O (coefficient = 0.68, P = 0.035) were associated with BMI. Rhesus factors were not associated with BMI (Table 4).

**Table 4:** Multiple linear regression of the factors associated with body mass index in pregnant women in eastern Sudan, 2022

Variables		Coefficient (standard error)	P-value
Age, years		0.07 (0.033)	0.028
Gravidity		0.25 (0.102	0.014
Residence	Urban	1.33 (0.412)	0.001
	Rural	Reference	
Education level	≥ secondary	Reference	0.612
	< secondary	0.23 (0.446)	
Occupation	Housewives	Reference	0.105
	Employed	1.28 (0.791)	
Blood groups	O	0.68(0.325)	0.035
	Other than O	Reference	
Rhesus factor	Positive	Reference	0.732
	Negative	–0.275 (0.802)	

## Discussion

To the best of the authors’ knowledge, this is the first study to explore the association between maternal obesity and blood groups. In this study, 22.0% of pregnant women were obese, and blood group O was associated with obesity and BMI among pregnant Sudanese women during early pregnancy. The results indicated a high prevalence (22.0%) of maternal obesity in eastern Sudan, comparable with previous findings from central Sudan (20.0–29.9) [3, 4]. This prevalence of maternal obesity (22.0%) was considerably higher than that reported in other countries, including Nigeria (10.7%) [32] and United Arab Emirates (6.1%) [11]. Marked variations in the prevalence of maternal obesity have been reported among African countries (6.5–50.7%) [2]. For example, a recent cross-sectional study involving317 pregnant women showed a high prevalence of maternal obesity (42.3%) in Cameroon [33].

The high prevalence of maternal obesity in Africa can be attributed to several factors, including low maternal education, poor understanding of the complications of maternal obesity, late initiation of antenatal care, and unhealthy lifestyles, including alcohol consumption [4, 33]. This high prevalence of maternal obesity could explain the poor maternal and perinatal outcomes reported in many countries, including Sudan [6, 10, 34].

In the present study, group O (58.5%) was the most common blood group, followed by group A (22.0%). This is similar to the results of our previous studies on pregnant women in central Sudan [14] and eastern Sudan [15].

Our previous cross-sectional study, which included 186 pregnant women in Khartoum, central Sudan, showed that women with blood group O were at higher risk of *H. pylori* IgG seropositivity [14]. Additionally, our previous study of 293 women delivering in eastern Sudan revealed that women with blood group O were at higher risk of placental malaria infection [15].

Compared with studies from different countries, a cross-sectional study on200 female university students in Faisalabad, Pakistan, reported a significant association between blood group O, obesity and BMI [17]. In a Turkish study involving 306 females (aged 18–65 years), females with blood groups A and AB were more likely to develop obesity than their counterparts with blood group O [19].

In contrast, a cross-sectional study that included114 students (47 females and 67 males) in Lahore, Pakistan, revealed no association between blood groups and BMI [21]. Similarly, across-sectional study on1,171 Saudi participants (575 men and 596 women age ≥ 15 years) reported no association between the prevalence of obesity or high BMI and blood groups [22]. The divergent findings of the present study could be due to the differences in the participants’ ages and genders, the distribution of blood groups in different populations, and the categorization of blood groups as Rhesus negative and positive. For example, a cross-sectional study that included 549,690 blood donors (34.6% women) attributed the influence of blood groups on BMI to gender (blood group B and blood group O), with a greater prevalence of obesity in women and a decreased prevalence of obesity in men [18]. In the present study, women with blood groups B and O had a significantly higher BMI. Such differences should encourage researchers to conduct more research on different population groups, especially pregnant women, as no studies have examined the association between maternal obesity and blood groups.

The findings of the present study showed no association between Rhesus factors and overweight, obesity, or BMI. Similarly, a case-control study involving 200 participants (100 patients and 100 controls) showed no association between Rhesus factors and COVID-19 in Sudanese patients suffering from different chronic diseases [16]. Moreover, a study in Turkey showed no association between Rhesus factors and obesity among adult females [19].

The mechanism(s) by which blood groups influence BMI remains unclear. However, the possibility that blood groups induce obesity through inflammatory mechanisms and genetic back grounds cannot be ruled out. Currently, there is a debate regarding managing obesity by inhibiting inflammatory mechanisms [35]. Such inhibition might contribute to preventing obesity and its complications, including cardiovascular diseases [35]. Although adiponectin (an adipokine secreted by adipocytes) has been reported to have a negative association with cancer, cardiovascular disease, and diabetes mellitus and is significantly increased by good nutrition [36], research is needed to evaluate adiponectin levels in different blood groups. Moreover, there is still debate regarding blood type-specific diets(i.e. each blood group is associated with certain food types) [37]. For example, a randomized crossover trial investigated the impact of daily consumption of myo-inositol hexaphosphate (phytate; InsP6) on serum levels of various inflammatory biomarkers, including adiponectin, in patients (n = 30) with type 2 diabetes mellitus. The results showed that patients who consumed InsP6 supplements for three months had higher levels of adiponectin and lower glycated hemoglobin (HbA1c) than patients who did not consume InsP6 [38]. Tirthani et al. examined the role of genetics and epigenetics in obesity, specifically family history and ethnicity [39]. Notably, variations of blood groups have been reported among different African ethnicities [40].

The current study was primarily conducted to assess the association between blood groups and maternal obesity. Besides blood group O, old age and urban residence were associated with maternal obesity and high BMI. This result is similar to those of previous studies in different countries, including Sudan [3, 4, 11, 32, 41]. We have discussed this in our previous works [3, 4, 11]. Further discussion of these factors is beyond the scope of the current paper.

Although the current study is the first to examine the association between blood groups and maternal obesity, there are some limitations that need to be acknowledged, and overcome in future studies. This was a cross-sectional study, this limits the ability to establish causality or determine the temporal relationship between blood groups, BMI, and obesity. Longitudinal studies or experimental designs would provide stronger evidence for causal relationships. Although our study had a large sample size, it focused on a single geographical region (eastern Sudan); therefore, it might not be large enough to represent the entire population of pregnant women in Sudan or other regions. In such kind of studies’ design, recall biases, cannot be rule out, however, in this study, several measurements were taken to minimize such biases, of them, the medical assistants were well trained to collect appropriate information, and all used devices were calibrated. The present study did not include a control group, which makes it difficult to compare the present results with other groups such as a non-pregnant population or pregnant women with different characteristics. Including of control group will explore more the relationship between blood groups, BMI, and obesity. However, in the present study, logistic regression analyses were performed to control for confounding factors. Moreover, the present study did not collect information socioeconomic status, dietary habits, or physical activity levels, which are known to influence obesity. These factors might act as potential confounding factors; therefore, collecting and controlling of such factors are essential to be taken into account in future studies to increase the validity of the results and the ability to attribute the observed associations solely to blood groups. Future studies are recommended to overcome such limitations.

## Conclusions

The results indicate that pregnant women in eastern Sudan are at risk of obesity. In this study, women with blood group O were at higher risk of maternal obesity and high BMI. Further research is needed to explore the mechanisms by which blood groups influence obesity.

## Data Availability

Data generated or analyzed during this study are included in this article and are available from the corresponding author upon reasonable request.

## Abbreviations

AOR: adjusted odds ratio
BMI: body mass index
CI: confidence interval
IQR: interquartile range
SPSS: Statistical Package for the Social Sciences
WHO: World Health Organization

## References

[1] World Health Organization, Obesity. and overweight. 2021. https://www.who.int/news-room/fact-sheets/detail/obesity-and-overweight, Acessed 2/11/2023.

[2] Onubi OJ, Marais D, Aucott L, Okonofua F, Poobalan AS (2016). Maternal obesity in Africa: a systematic review and meta-analysis. J Public Heal (United Kingdom).

[3] Elmugabil A, Rayis DA, Abdelmageed RE, Adam I, Gasim GI (2017). High level of hemoglobin, white blood cells and obesity among Sudanese women in early pregnancy: a cross-sectional study. Futur Sci OA.

[4] Rayis DA, Abbaker AO, Salih Y, Diab TE, Adam I (2010). Epidemiology of underweight and overweight- obesity among term pregnant Sudanese women. BMC Res Notes.

[5] Ellis JA, Brown CM, Barger B, Carlson NS (2019). Influence of maternal obesity on labor induction: a systematic review and meta-analysis. J Midwifery Women’s Heal.

[6] Rayis DA, Abbaker AO, Salih Y, Adam I (2011). Obesity and pregnancy outcome in Khartoum, Sudan. Int J Gynecol Obstet.

[7] Taha Z, Hassan AA, Wikkeling-Scott L, Papandreou D (2019). Prevalence and associated factors of caesarean section and its impact on early initiation of breastfeeding in Abu Dhabi, United Arab Emirates. Nutrients.

[8] Moftakhar L, Solaymani-Dodaran M, Cheraghian B (2018). Role of obesity in gestational Hypertension in primigravidae women: a case control study in Shadegan, Iran. Med J Islam Repub Iran.

[9] Linder T, Eder A, Monod C, Rosicky I, Eppel D, Redling K et al. Impact of prepregnancy overweight and obesity on treatment modality and pregnancy outcome in women with gestational Diabetes Mellitus. Front Endocrinol (Lausanne). 2022;13 May:799625. 10.3389/fendo.2022.799625 PMC9160363 35663318

[10] Reed J, Case S, Rijhsinghani A. Maternal obesity: perinatal implications. SAGE Open Med. 2023;11. 10.1177/20503121231176128. 10.1177/20503121231176128 PMC10233600 37275842

[11] Taha Z, Hassan AA, Papandreou D (2022). Epidemiology of pre-pregnancy body mass index (BMI) among mothers in Abu Dhabi, the United Arab Emirates. Front Glob Women’s Heal.

[12] Chandra T, Gupta A (2012). Association and distribution of Hypertension, obesity and ABO blood groups in blood donors. Iran J Pediatr Hematol Oncol.

[13] Elmugabil A, Rayis DA, Ahmed MA, Adam I, Gasim GI (2016). O blood group as risk factor for preeclampsia among Sudanese women. Open Access Maced J Med Sci.

[14] Gasim GI, Elmugabil A, Hamdan HZ, Rayis DA, Adam I (2017). O blood group as a risk factor for helicobacter pylori IgG seropositivity among pregnant Sudanese women. Clin Pract.

[15] Adam I, Babiker S, Mohmmed AA, Salih MM, Prins MH, Zaki ZM (2007). ABO blood group system and placental Malaria in an area of unstable Malaria transmission in eastern Sudan. Malar J.

[16] Mohamed MF, Ahmad A, Elmahi OM, Babker AM, Waggiallah HA (2021). Susceptibility of blood groups Infection with covid-19 Disease among Sudanese patients suffering from different chronic Diseases. Pakistan J Biol Sci.

[17] Jawed S, Atta K, Tariq S, Amir F (2018). How good is the obesity associated with blood groups in a cohort of female university going students?. Pakistan J Med Sci.

[18] Flor CR, Gomes Moura IC, Baldoni AO, Loureiro P, Teixeira CM, Carneiro-Proietti AB (2020). Obesity and ABO blood group: is there an association?. Obes Med.

[19] Eren C, Çeçen S (2019). An analysis on the association between ABO and rh blood groups with obesity. Proc Natl Acad Sci India Sect B - Biol Sci.

[20] Siddiqui N, Soni A, Khan S (2019). Association of ABO blood types and novel obesity markers in healthy adolescents. J Educ Health Promot.

[21] Khan QU, Zaffar S, Zia MR, Aftab M, Nadeem N, Hafeez F (2021). Frequency of ABO blood groups and its relationship with body mass index in students of a medical college in lahore. Pakistan J Med Heal Sci.

[22] Alwasaidi TA, Alrasheed SK, Alhazmi RA, Alfraidy OB, Jameel MA, Alandijani AA (2017). Relation between ABO blood groups and obesity in a Saudi Arabian population. J Taibah Univ Med Sci.

[23] Yang Y, Cai Z, Zhang J (2021). The effect of prepregnancy body mass index on maternal micronutrient status: a meta-analysis. Sci Rep.

[24] Rayis DA, Karar A, Alshareef SA, Eltayeb R, Adam I (2023). Early pregnancy serum levels of folate and vitamin B12 in overweight and obese women in Khartoum, Sudan. Trans R Soc Trop Med Hyg.

[25] Abbas W, Adam I, Rayis DA, Hassan NG, Lutfi MF (2017). A higher rate of iron Deficiency in obese pregnant Sudanese women. Open Access Maced J Med Sci.

[26] UNICEF. Situation in Gedaref. 2022. https://www.unicef.org/sudan/media/8651/file/Gedarif.pdf. Acesed 2/11/2023.

[27] World Health Organization. Obesity: preventing and managing the global epidemic. Report of a WHO consultation. https://apps.who.int/iris/handle/10665/42330. Acesed 2/11/2023. 11234459

[28] Abbas AA (2017). Frequency of ABO and rh D blood groups among Sudanese blood donors attending Central Blood Bank in Wad Medani, Gezira State, Sudan. Int J Med Res Prof.

[29] Li HY, Guo K. Blood group testing. Front Med. 2022;9 February:827619. 10.3389/fmed.2022.827619 PMC8873177 35223922

[30] Dean AG, Sullivan KM, Soe MM, OpenEpi. Open Source Epidemiologic Statistics for Public Health, Version. www.OpenEpi.com, updated 2013/04/06. Acessed 20/08/2023.

[31] Ali EA, Almugabil A, Salim A, Rayis DA, Adam I (2017). The effect of interpregnancy interval on obesity/overweight among women in the first trimester of pregnancy. Int J Gynaecol Obstet.

[32] Chigbu C, Aja L (2011). Obesity in pregnancy in southeast Nigeria. Ann Med Health Sci Res.

[33] Agwara EO, Tendongfor N, Jaja PT, Choy AM, Egbe TO. Prevalence and pregnant women’s knowledge of maternal obesity and excessive gestational weight gain among women attending antenatal care in Fako Division, Cameroon. Pan Afr Med J. 2023;44. 10.11604/pamj.2023.44.2.36592. 10.11604/pamj.2023.44.2.36592 PMC9935657 36818033

[34] Elhassan E, Hassan A, Haggaz A, Ali A, Adam I (2010). Epidemiology of maternal mortality and poor perinatal outcomes in different regions of Sudan. Gezira J Heal Sci.

[35] Ellulu MS, Patimah I, Khaza’ai H, Rahmat A, Abed Y (2017). Obesity & inflammation: the linking mechanism & the Complications. Arch Med Sci.

[36] Khoramipour K, Chamari K, Hekmatikar AA, Ziyaiyan A, Taherkhani S, Elguindy NM (2021). Diseases, and effects of nutrition. Nutrients.

[37] Shmerling RH. Diet not working? Maybe its not your type what’s the blood type diet? 2022. https://www.health.harvard.edu/blog/diet-not-working-maybe-its-not-your-type-2017051211678.

[38] Sanchis P, Calvo P, Pujol A, Rivera R, Berga F, Fortuny R (2023). Daily phytate intake increases adiponectin levels among patients with Diabetes type 2: a randomized crossover trial. Nutr Diabetes.

[39] Smith ENL, Chandanathil M, Millis RM (2023). Epigenetic mechanisms in obesity: broadening our understanding of the Disease. Cureus.

[40] Doku GN, Agbozo WK, Annor RA, Mawudzro PE, Agbeli EE (2022). Frequencies and ethnic distribution of ABO and RhD blood groups in the Volta region of Ghana, towards effective blood bank services. Afr Health Sci.

[41] Bliznashka L, Jeong J, Jaacks LM (2023). Maternal and paternal employment in agriculture and early childhood development: a cross-sectional analysis of demographic and Health Survey data. PLOS Glob Public Heal.

